# Intercellular adhesion molecule-1 augments myoblast adhesion and fusion through homophilic *trans*-interactions

**DOI:** 10.1038/s41598-017-05283-3

**Published:** 2017-07-11

**Authors:** Francis X. Pizza, Ryan A. Martin, Evan M. Springer, Maxwell S. Leffler, Bryce R. Woelmer, Isaac J. Recker, Douglas W. Leaman

**Affiliations:** 1 School of Exercise and Rehabilitation Sciences, University of Toledo, Toledo, Ohio USA; 2 Department of Biological Sciences, University of Toledo, Toledo, Ohio USA; 3 Wright State University, 4035 Colonel Glenn Hwy., Suite 300, Beavercreek, OH 45431 USA

## Abstract

The overall objective of the study was to identify mechanisms through which intercellular adhesion molecule-1 (ICAM-1) augments the adhesive and fusogenic properties of myogenic cells. Hypotheses were tested using cultured myoblasts and fibroblasts, which do not constitutively express ICAM-1, and myoblasts and fibroblasts forced to express full length ICAM-1 or a truncated form lacking the cytoplasmic domain of ICAM-1. ICAM-1 mediated myoblast adhesion and fusion were quantified using novel assays and cell mixing experiments. We report that ICAM-1 augments myoblast adhesion to myoblasts and myotubes through homophilic *trans*-interactions. Such adhesive interactions enhanced levels of active Rac in adherent and fusing myoblasts, as well as triggered lamellipodia, spreading, and fusion of myoblasts through the signaling function of the cytoplasmic domain of ICAM-1. Rac inhibition negated ICAM-1 mediated lamellipodia, spreading, and fusion of myoblasts. The fusogenic property of ICAM-1-ICAM-1 interactions was restricted to myogenic cells, as forced expression of ICAM-1 by fibroblasts did not augment their fusion to ICAM-1+ myoblasts/myotubes. We conclude that ICAM-1 augments myoblast adhesion and fusion through its ability to self-associate and initiate Rac-mediated remodeling of the actin cytoskeleton.

## Introduction

Cell-to-cell interactions associated with the formation of muscular tissue (myogenesis) are fundamentally important during embryogenesis (developmental myogenesis), and in restoring structure and function to skeletal muscle injured by physical activity, trauma, or disease (regenerative myogenesis). Such interactions are of particular importance in the initial stage of myogenesis when muscle precursor cells called myoblasts adhere to each other and fuse to form myotubes, as well as when myoblasts adhere and fuse with myotubes^1, 2^. Myotubes also add nuclei through myotube-myotube fusion, particularly in the later stage of myogenesis^3, 4^. In injured muscle, cell-to-cell interactions of myogenesis give rise to central nucleated (regenerating) myofibers, which restore structure and function to injured muscle by hypertrophying into normal myofibers^1, 5^.

Membrane structures that mediate myoblast adhesion to myoblasts and myotubes, and mechanisms through which such adhesion triggers fusion, are not well understood. Cell adhesion molecules of cadherin (e.g., M- and N-cadherin) and immunoglobulin (e.g., NCAM/CD56 and VCAM-1/CD106) families^2^, as well as other membrane proteins (e.g., myoferlin, MOR23, and myomaker) that are constitutively expressed by myogenic cells^6–8^ facilitate cell-to-cell interactions of myogenesis through their adhesive and/or signaling properties. Specifically, homophilic *trans*-interactions for cadherins and NCAM^9–12^, and heterophilic *trans*-interactions between VCAM-1 and VLA-4 (CD49d)^13^, myoferlin and phospholipids^7^, have been reported to serve as mechanisms through which myoblasts adhere to myoblasts and/or myotubes. Such adhesion can regulate subsequent events of myogenesis through activation of signaling molecules (e.g., p38 MAPK, GTPase Rac, and Akt/mTOR) and secretion of cytokines (e.g., IL-4) that promote migration, differentiation, actin polymerization, nuclear positioning, and/or protein synthesis in myoblasts and/or myotubes^3, 11, 14–16^. Due to an apparent functional redundancy between and within families of cell adhesion molecules, conflicting findings have been reported on the contribution of cadherins, NCAM, VCAM-1, and VLA-4 to developmental myogenesis^1, 2^.

In contrast to developmental myogenesis, regenerative myogenesis occurs when cells (e.g., macrophages) and cytokines (e.g., TNF-α) of the inflammatory response are accumulating within skeletal muscle^17, 18^, as well as when satellite cells/myoblasts, regenerating myofibers, and/or myofibers are expressing intercellular adhesion molecule-1 (ICAM-1/CD54)^19–23^, a member of the immunoglobulin superfamily of adhesion molecules that is not normally expressed by myogenic cells. Induced expression of ICAM-1 by myoblasts and myotubes also occurs *in vitro* after cytokine treatment^19, 24–27^. Importantly, ICAM-1 expression by myogenic cells contributes to regenerating myofiber formation within overloaded muscles^19^, and forced expression of ICAM-1 by cultured myoblasts augments myoblast-myoblast adhesion, myotube formation, myonuclear accretion, and myotube size, without influencing myoblast proliferation or differentiation^4^. Underlying mechanisms through which ICAM-1 augments the adhesive and fusogenic properties of myoblasts remains to be determined.

The objective of the study was to elucidate mechanisms through which ICAM-1 expression by myoblasts augments their adhesion and fusion to myoblasts and myotubes. As myoblasts do not express established ligands for ICAM-1 (e.g., CD11a and CD11b)^4, 26, 27^, we tested the hypothesis that homophilic *trans*-interactions serve as a mechanism through which ICAM-1 augments the adhesion of myoblasts to myoblasts and myotubes. We also tested the hypothesis that homophilic *trans*-interactions for ICAM-1 triggers myoblast fusion through the signaling function of the cytoplasmic domain of ICAM-1, and a mechanism involving Rac-mediated remodeling of the actin cytoskeleton.

## Results

### ICAM-1 Does Not Influence Myoblast Motility

Given that myoblasts migrate towards neighboring myoblasts and myotubes prior to their fusion^3, 16^, we explored the possibility that ICAM-1 augments myoblast fusion^4^ by enhancing their motility. Motility of sub-confluent cultures of myoblasts stably transfected with an empty vector (EV) or an ICAM-1 plasmid (ICAM-1+) was quantified via time-lapse microscopy at 1 d of differentiation. The mean accumulated distance, velocity (Figure S1), as well as displacement and directionality (data not reported) of migratory paths were similar for EV and ICAM-1+ myoblasts. These findings demonstrate that ICAM-1 does not influence myoblast motility.

### Homophilic Binding of ICAM-1

**Figure 1:**
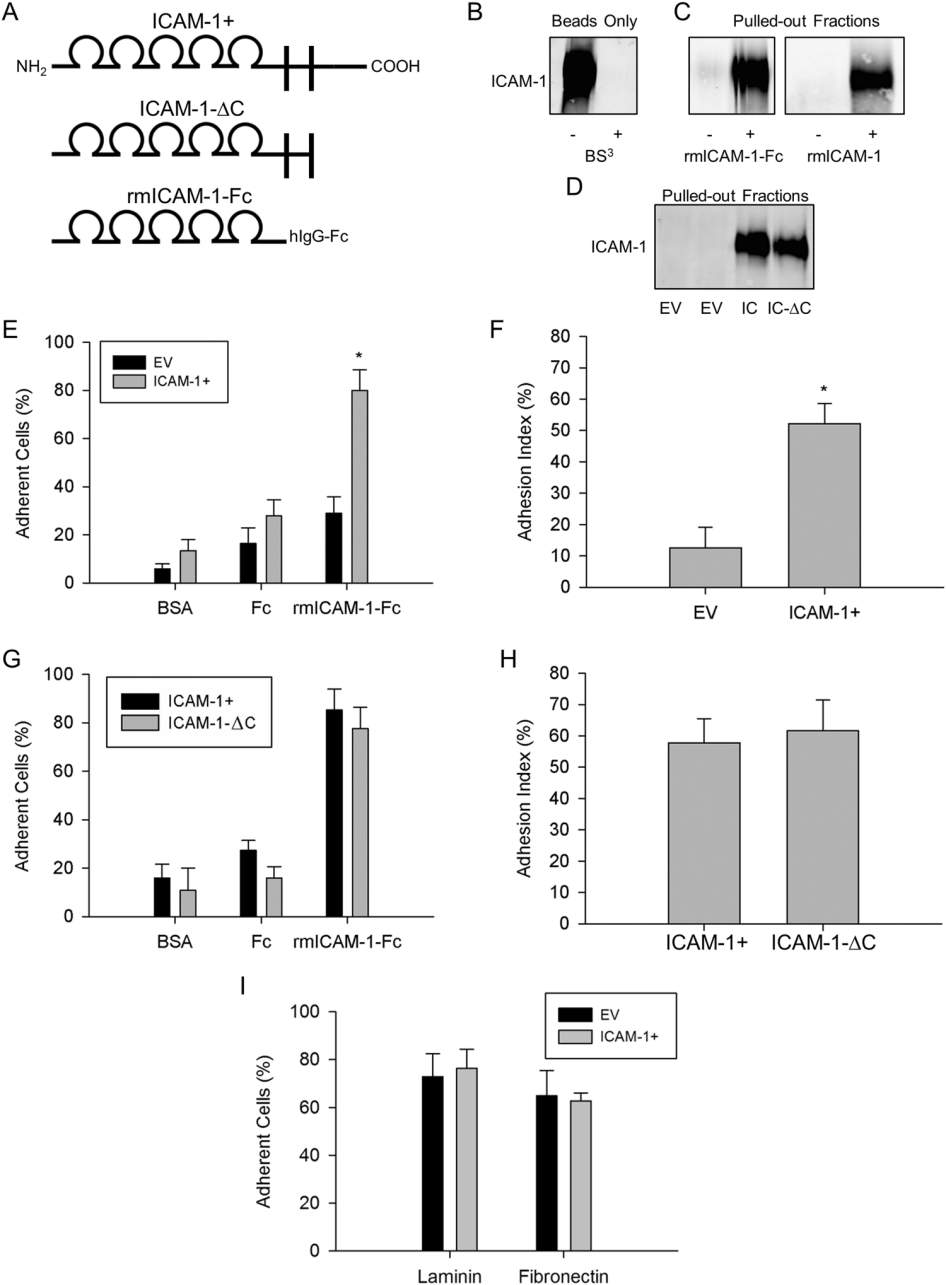
ICAM-1-ICAM-1 interactions in myoblast adhesion. (**A**) Structure of ICAM-1 in ICAM-1+ and ICAM-1-∆C cells, as well as rmICAM-1-Fc. Cartoon depicts the 5 extracellular IgG-like domains, transmembrane segment, and the cytoplasmic domain of ICAM-1. (**B**) Western blot for ICAM-1 in beads coated with rmICAM-1-Fc. BS^3^ was used to covalently link rmICAM-1-Fc to beads. The cropped area corresponds to 150–100 kDa and the ICAM-1 band appeared at ~110 kDa. (**C**) Beads crosslinked with ICAM-1 were incubated with PBS-T, rmICAM-1-Fc or rmICAM-1. ICAM-1 was detected in pulled-out fractions via western blotting. (**D**) Western blots for ICAM-1 in pulled-out fractions of EV, ICAM-1+ (IC), and ICAM-1-∆C (IC-∆C) myoblasts. (**E**) The percentage of EV and ICAM-1+ myoblast that adhere to bovine serum albumin (BSA), rhIgG1-Fc (Fc), or rmICAM-1-Fc. *Higher for ICAM-1+ compared to EV myoblasts for rmICAM-1-Fc (interaction effect; p < 0.005). (**F**) Adhesion index for EV and ICAM-1+ myoblasts. *Higher (p < 0.005) for ICAM-1+ compared to EV myoblasts. (**G**) The percentage of ICAM-1+ and ICAM-1-∆C myoblasts that adhere to BSA, Fc, and rmICAM-1-Fc. (**H**) Adhesion index for ICAM-1+ and ICAM-1-∆C myoblasts. (**I**) The percentage of EV and ICAM-1+ myoblasts that adhere laminin and fibronectin (p = 0.56). n = 4 replicates for each experimental condition and data set.

We began testing the involvement of homophilic *trans*-interactions for ICAM-1 in myoblast adhesion and subsequent fusion by determining the extent to which the extracellular domain of ICAM-1 can bind to itself. Recombinant murine ICAM-1 dimerized to the Fc portion of human IgG1 (rmICAM-1-Fc; Fig. 1A), a non-chimeric form of ICAM-1 (rmICAM-1), and proteins in cell lysates were used as prey, and rmICAM-1-Fc cross-linked to magnetic beads was used as bait. Cross-linking was effective as indicated by the lack of detection of ICAM-1 in beads coated with rmICAM-1-Fc and treated with bis(sulfosuccinimidyl) suberate (BS)^3^ (Figs 1B and S2B). Both recombinant forms of ICAM-1 were found to bind beads cross-linked with rmICAM-1-Fc (Figs 1C and S2C). No ICAM-1 was detected in pulled-out fractions of EV myoblasts (Figs 1D and S2D), which do not express ICAM-1 (Figure S3)^4^. In contrast, a prominent ICAM-1 band was observed in pulled-out fractions of ICAM-1+ myoblasts, as well as in myoblasts that express the extracellular and transmembrane domains, but not the cytoplasmic domain of ICAM-1 (ICAM-1-∆C). These findings indicate that the extracellular domain of ICAM-1 is capable of binding to itself.

### Homophilic Binding of ICAM-1 Mediates Myoblast Adhesion

We quantified the adhesion of myoblasts treated with differentiation medium for 1 d (fusion competent myoblasts) to wells coated with rmICAM-1-Fc to determine the extent to which ICAM-1-ICAM-1 interactions could support myoblast adhesion. As expected, the percentage of myoblasts adherent to wells coated with bovine serum albumin (BSA) or recombinant human IgG1-Fc (Fc) was low (Fig. 1E), and similar between EV and ICAM-1+ myoblasts. In contrast, the percentage of myoblasts that adhered to wells coated with rmICAM-1-Fc was 2.8 fold higher for ICAM-1+ compared to EV myoblasts. The adhesion index, which takes into account myoblast adhesion to rmICAM-1-Fc and Fc, was 4.2 fold higher for ICAM-1+ compared to EV myoblasts (Fig. 1F). These findings are consistent with the hypothesis that homophilic *trans*-interactions for ICAM-1 augment adhesive interactions between opposing myogenic cells.

### Cytoplasmic Domain of ICAM-1 Does Not Contribute to Myoblast Adhesion to ICAM-1

To test the contribution of the cytoplasmic domain of ICAM-1 to myoblast adhesion, we counted and compared the adhesion of fusion competent ICAM-1+ and ICAM-1-∆C myoblasts to wells coated with rmICAM-1-Fc. The percentage of myoblasts that adhered to wells coated with BSA, Fc and rmICAM-1-Fc, as well as the adhesion index, were similar for ICAM-1+ and ICAM-1-∆C myoblasts (Fig. 1G and H). These findings demonstrate that the signaling function of the cytoplasmic domain of ICAM-1 does not contribute to myoblast adhesion to ICAM-1.

### ICAM-1 Does Not Influence Myoblast Adhesion to Laminin and Fibronectin

To explore the influence of ICAM-1 on myoblast adhesion to components of the extracellular matrix, we counted and compared the adhesion of fusion competent EV and ICAM-1+ myoblasts to wells coated with laminin and fibronectin. The percentage of EV and ICAM-1+ myoblasts that adhered to laminin and fibronectin coated wells was similar (Fig. 1I). The extent to which ICAM-1 influences myoblast adhesion to other components of the extracellular matrix (e.g., collagen) remains to be determined.

### ICAM-1 Augments Myoblast-Myoblast Adhesion Through Homophilic Interactions

Using a cell suspension aggregation assay, we previously demonstrated that myoblast-myoblast adhesion was 2–3 fold greater for ICAM-1+ compared to EV myoblasts^4^. We also reported that the extracellular domain of ICAM-1 contributes to ICAM-1 mediated myoblast-myoblast adhesion, as antibody neutralization of ICAM-1 inhibited myoblast aggregation^4^.

**Figure 2:**
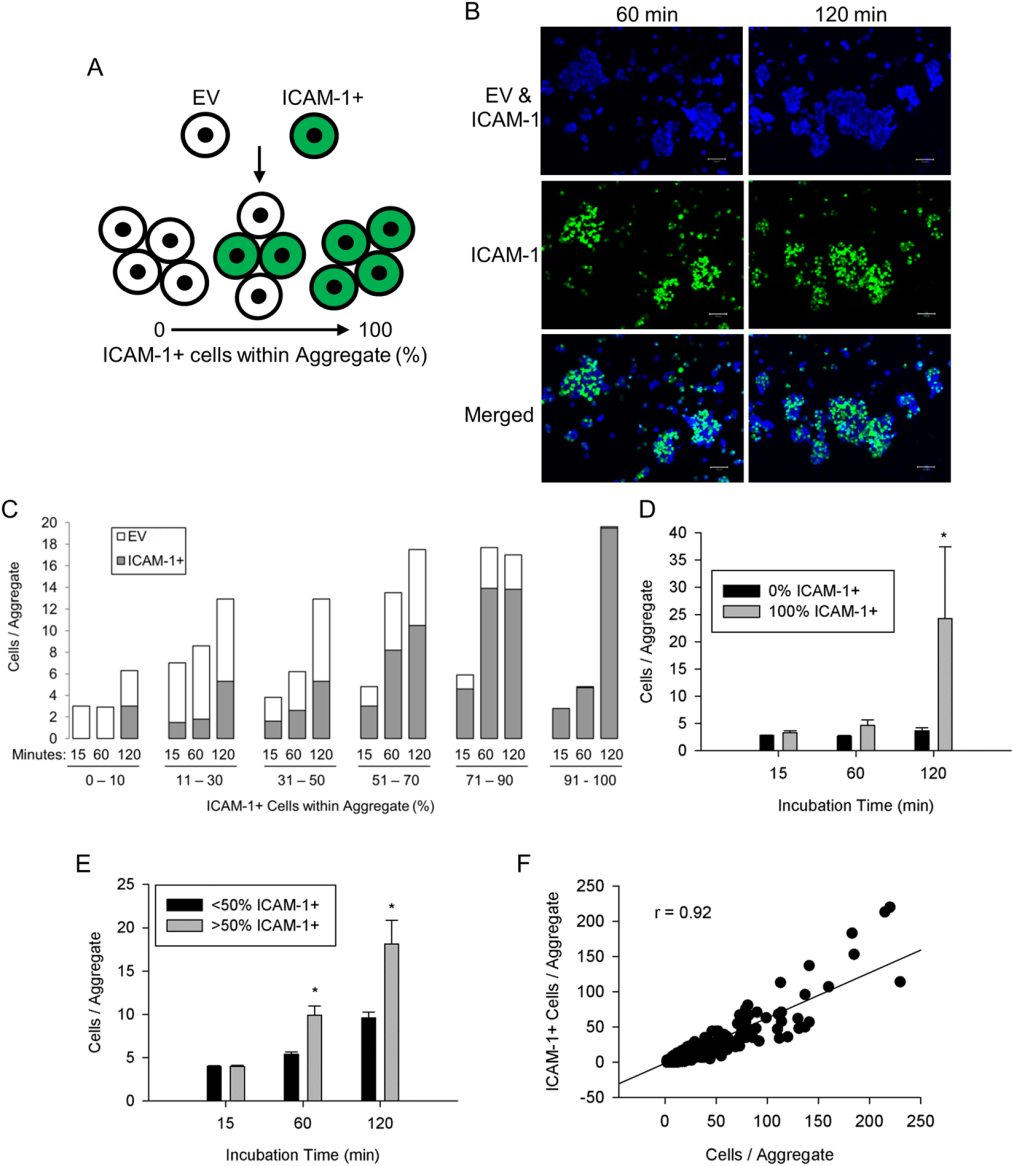
ICAM-1-ICAM-1 interactions in myoblast-myoblast adhesion. (**A**) ICAM-1+ myoblasts were labeled with CellTracker™ Green, mixed with EV myoblasts in equal number, and the number of EV and ICAM-1+ myoblasts within aggregates was quantified. (**B**) Images of wheat germ agglutinin (WGA; blue), which delineated both EV and ICAM-1+ myoblasts, and ICAM-1+ myoblasts (green) at 60 and 120 min of incubation (scale bar = 100 µm). (**C**) Frequency distribution of the average number of EV and ICAM-1+ myoblasts within aggregates as a function of the percentage of ICAM-1+ myoblasts within the aggregate. The number of aggregates analyzed was 1818, 1336, and 948 at 15, 60, and 120 min of incubation, respectively. (**D**) The average number of myoblasts/aggregate for aggregates that contained only EV (0% ICAM-1+) or ICAM-1+ (100% ICAM-1+) myoblasts. *Higher for 100% ICAM-1+ compared to 0% ICAM-1+ at 120 min of incubation (interaction effect; p < 0.001). (**E**) The average number of myoblasts/aggregate for aggregates that contained primarily ICAM-1+ (>50% ICAM-1+) or EV myoblasts (≤50% ICAM-1+). *Higher for >50% ICAM-1+ compared to ≤ 50% ICAM-1+ at 60 and 120 min of incubation (interaction effect; p < 0.001). (**F**) Scatter plot of the number of ICAM-1+ myoblasts/aggregate vs. the number myoblasts/aggregate (n = 2232 aggregates at 60 and 120 min of incubation). A high Pearson-product moment correlation was observed (r = 0.92; p < 0.001).

In the present study, we quantified myoblast aggregation after mixing fusion competent EV and ICAM-1+ myoblasts in equal number to determine the extent to which homophilic binding of ICAM-1 facilitates myoblast-myoblast adhesion (Fig. 2A and B). Importantly, 50.4% of all the cells counted (n = 39,908) were ICAM-1+ myoblasts. Given that EV and ICAM-1+ myoblasts were mixed in equal number, an ICAM-1 independent mechanism for myoblast-myoblast adhesion would result in the formation of aggregates that contain approximately the same number of each cell type. On the other hand, homophilic *trans*-interactions for ICAM-1 would result in the formation of large aggregates that contain primarily ICAM-1+ myoblasts.

Frequency distribution plots show a progressive rise in the average number of myoblasts within an aggregate, with increasing percentage of ICAM-1+ myoblasts within aggregates (Fig. 2C). A rightward shift in the distribution occurred over time, particularly for aggregates that contained almost exclusively ICAM-1+ myoblasts (91–100% ICAM-1+). For these aggregates, the increased size over time was accompanied by a comparable reduction in the number of aggregates (~2 and 12 fold at 60 and 120 min, respectively compared to 15 min). At the end of the assay, the average number of myoblasts within an aggregate was 6 fold greater for aggregates that contained only ICAM-1+ myoblasts compared to those that contained only EV myoblasts (Fig. 2D). Rightward shifts in frequency distributions indicate that ICAM-1+ myoblasts adhered to each other to form large aggregates over time.

As 65% of the aggregates contained EV and ICAM-1+ myoblasts, we partitioned aggregates into 2 groups based on the percentage of myoblasts within an aggregate that were ICAM-1+. The average number of myoblasts within an aggregate at 60 and 120 min was 2 fold higher for aggregates that contained primarily ICAM-1+ myoblasts (>50% ICAM-1+), compared to aggregates in which ICAM-1+ myoblasts were the minority (≤50% ICAM-1+) (Fig. 2E). Importantly, the number of ICAM-1+ myoblasts within an aggregate was highly correlated to the number of myoblasts within the aggregate (Fig. 2F). Collectively, our findings demonstrate that homophilic *trans*-interactions for ICAM-1 serve as a mechanism for ICAM-1 mediated myoblast-myoblast adhesion.

### Homophilic Binding of ICAM-1 Facilitates Myogenic Cell Fusion

**Figure 3:**
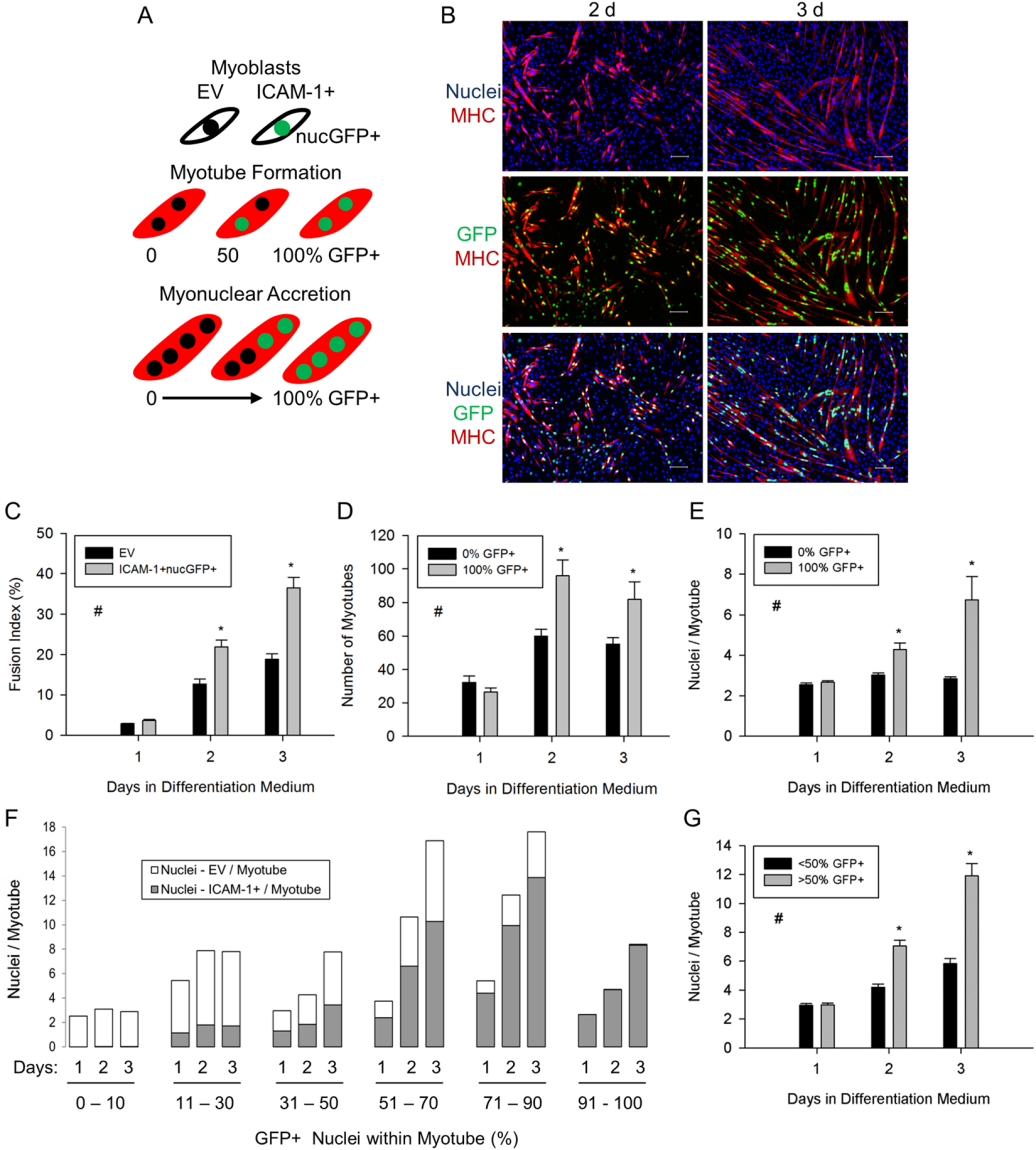
ICAM-1-ICAM-1 interactions in myoblast fusion. (**A**) EV and ICAM-1+ nucGFP+ myoblasts were mixed in equal number, and myotube indices were quantified through 3 d of differentiation. MHC expression (red) was used a marker of myogenic cell differentiation. (**B**) Images of DAPI+ nuclei (blue) of EV and ICAM-1+ nucGFP+ myoblasts, MHC (red), and nuclei of ICAM-1+ nucGFP+ myoblasts (green) at 2 and 3 d of differentiation (scale bar = 100 µm). (**C**) Fusion index for EV and ICAM-1+ nucGFP+ myoblasts. ^#^Higher for ICAM-1+ nucGFP+ compared to EV cells throughout 3 d of differentiation (main effect for cell line; p < 0.001). *Higher for ICAM-1+ nucGFP+ compared to EV cells at indicated day of differentiation (interaction effect; p < 0.001). (**D** and **E**) Myotube number (**D**) and the average number of nuclei within myotubes (**E**) that contained nuclei from only EV (0% GFP+) or ICAM-1+ nucGFP+ (100% GFP+) myoblasts. ^#^Higher for 100% GFP+ compared to 0% GFP+ throughout 3 d of differentiation (main effect for cell line; p < 0.001). *Higher for 100% GFP+ compared to 0% GFP+ at indicated day of differentiation (interaction effect; p < 0.05). (**F**) Frequency distribution of the average number of nuclei from EV and ICAM-1+ nucGFP+ myoblasts within individual myotubes as a function of the percentage of GFP+ nuclei within a myotube. The number of myotubes analyzed was 425, 1845, and 1741 at 1, 2, and 3 d of differentiation, respectively. (**G**) The average number of nuclei/myotube for myotubes that contained nuclei primarily from ICAM-1+ nucGFP+ (>50% GFP+) or EV myoblasts (≤50% GFP+). ^#^Higher for >50% GFP+ compared to ≤50% GFP+ throughout 3 d of differentiation (main effect for cell line; p < 0.005). *Higher for >50% GFP+ compared to ≤50% GFP+ at indicated day of differentiation (interaction effect; p < 0.005). n = 4–6 replicates at each day of differentiation.

To determine the extent to which homophilic *trans*-interactions for ICAM-1 augment myoblast fusion, we mixed an equal number of EV myoblasts with myoblasts that express ICAM-1 and green fluorescent protein (GFP) in the nucleus (ICAM-1+ nucGFP+) (Fig. 3A). Importantly, 49.1% of the total nuclei counted (n = 231,181) in mixed cultures were nuclei from ICAM-1+ nucGFP+ myoblasts. The percentage of GFP+ nuclei (i.e., [nucGFP+/total nuclei] X 100) in mixed cultures at each day of differentiation was similar (standard deviation for 1–5 d of differentiation = 6.7%).

We reasoned that if homophilic binding of ICAM-1 triggers myoblast-myoblast fusion, then the number of myotubes that contain nuclei from only ICAM-1+ nucGFP+ myoblasts (100% GFP+) would be greater than the number of myotubes that contain nuclei from only EV myoblasts (0% GFP+). If homophilic binding of ICAM-1 serves as a mechanism for myonuclear accretion, then myonuclear number would be greater for myotubes that contain nuclei from only ICAM-1+ nucGFP+ myoblasts compared to myotubes that contained nuclei from only EV myoblasts, and hybrid myotubes would contain nuclei primarily from ICAM-1+ nucGFP+ myoblasts.

The fusion index, which reflects myoblast-myoblast, myoblast-myotube, and myotube-myotube fusion, was 1.7–2.0 fold higher for ICAM-1+ nucGFP+ compared to EV myoblasts at 2 and 3 d of differentiation (Fig. 3B and C), which is consistent with our prior work^4^. At 2 and 3 d of differentiation, the number of myotubes that contained nuclei from only ICAM-1+ nucGFP+ myoblasts was 1.5–2.5 fold higher compared to the number of myotubes that contained nuclei from only EV myoblasts (Fig. 3D). At 2 d of differentiation, the number of nascent myotubes (i.e., binucleated MHC+ cells) that contained nuclei from only ICAM-1+ nucGFP+ myoblasts was 45% higher than the number of nascent myotubes containing only EV nuclei (data not reported). Interestingly, the number of nascent hybrid myotubes at 2 d of differentiation was 21% higher compared to nascent myotubes containing only EV nuclei, suggesting that ICAM-1 expression by myoblasts augments their fusion with myoblasts that do not express ICAM-1. Collectively, these findings demonstrate that homophilic *trans*-interactions for ICAM-1 serve as mechanism through which ICAM-1 augments myotube formation^4^.

A contribution of ICAM-1-ICAM-1 interactions in myonuclear accretion was apparent in several data sets. At 2 and 3 d of differentiation, the number of nuclei within a myotube was 1.5–2.5 fold higher for myotubes that contained nuclei from only ICAM-1+ nucGFP+ myoblasts, compared to myotubes that contained nuclei from only EV myoblasts (Fig. 3E). As ~70% of the myotubes formed through 3 d of differentiation were hybrid myotubes, we performed frequency distribution analysis to further establish the involvement of ICAM-1-ICAM-1 interactions in myonuclear accretion. A progressive rise in myonuclear number was observed with increasing percentage of nuclei from ICAM-1+ nucGFP+ myoblasts within a myotube (Fig. 3F). Furthermore, the number of nuclei/myotube at 2 and 3 d of differentiation was 1.7–2.0 fold higher for myotubes that contained nuclei primarily from ICAM-1+ nucGFP+ myoblasts (>50% GFP+), compared to myotubes that contained nuclei primarily from EV myoblasts (≤50% GFP+) (Fig. 3G). These findings demonstrate that homophilic *trans*-interactions for ICAM-1 serve as mechanism through which ICAM-1 augments myoblast-myotube and/or myotube-myotube fusion^4^.

### ICAM-1 Does Not Influence Directed Migration of Myoblasts

**Figure 4:**
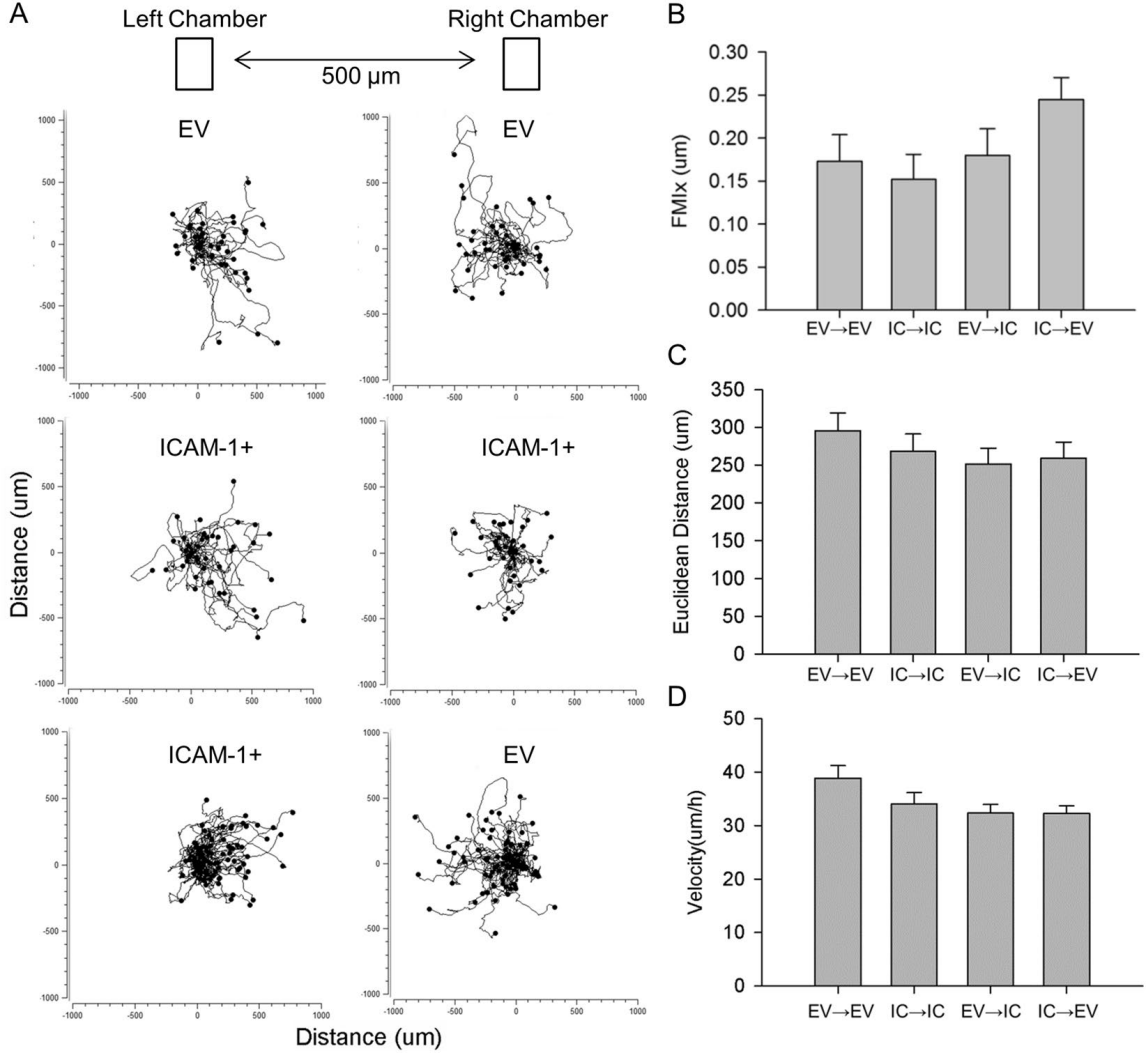
Directed migration of myoblasts. EV or ICAM-1+ myoblasts were seeded into the left and/or right chamber, treated with differentiation medium for 1 d, and their migratory paths were tracked for 20 h after removing the chamber from wells. (**A**) Migratory paths of EV and ICAM-1+ (IC) myoblasts towards EV or ICAM-1+ myoblasts. (**B**–**D**) FMI_x_ (B; p = 0.12), Euclidean distance (**C**), and velocity (**D**) of migratory paths. A total of 80 myoblasts for each cell line were analyzed in 4 independent experiments.

We explored the possibility that the myotube formation and myonuclear accretion resulting from ICAM-1-ICAM-1 interactions (Fig. 3D and E) was the result of an enhanced migration of ICAM-1+ myoblasts towards other ICAM-1+ cells. This was accomplished by quantifying migratory paths of ICAM-1+ myoblasts towards ICAM-1+ or EV myoblasts using 2 chamber inserts (Fig. 4A). As indicated by the forward migratory index on the x-coordinate (FMI_x_), the migration of ICAM-1+ myoblasts towards ICAM-1+ myoblasts was similar to their migration towards EV myoblasts (Fig. 4B). The FMI_x_ for EV myoblasts towards EV and ICAM-1+ myoblasts was also similar. No differences were observed in Euclidean distance (Fig. 4C) or velocity (Fig. 4D) of migratory paths between the experimental conditions. These findings demonstrate that ICAM-1 does not influence the migration of myoblasts towards other myoblasts.

### ICAM-1 Expression by Fibroblasts Does Not Augment Their Myogenic Conversion

**Figure 5:**
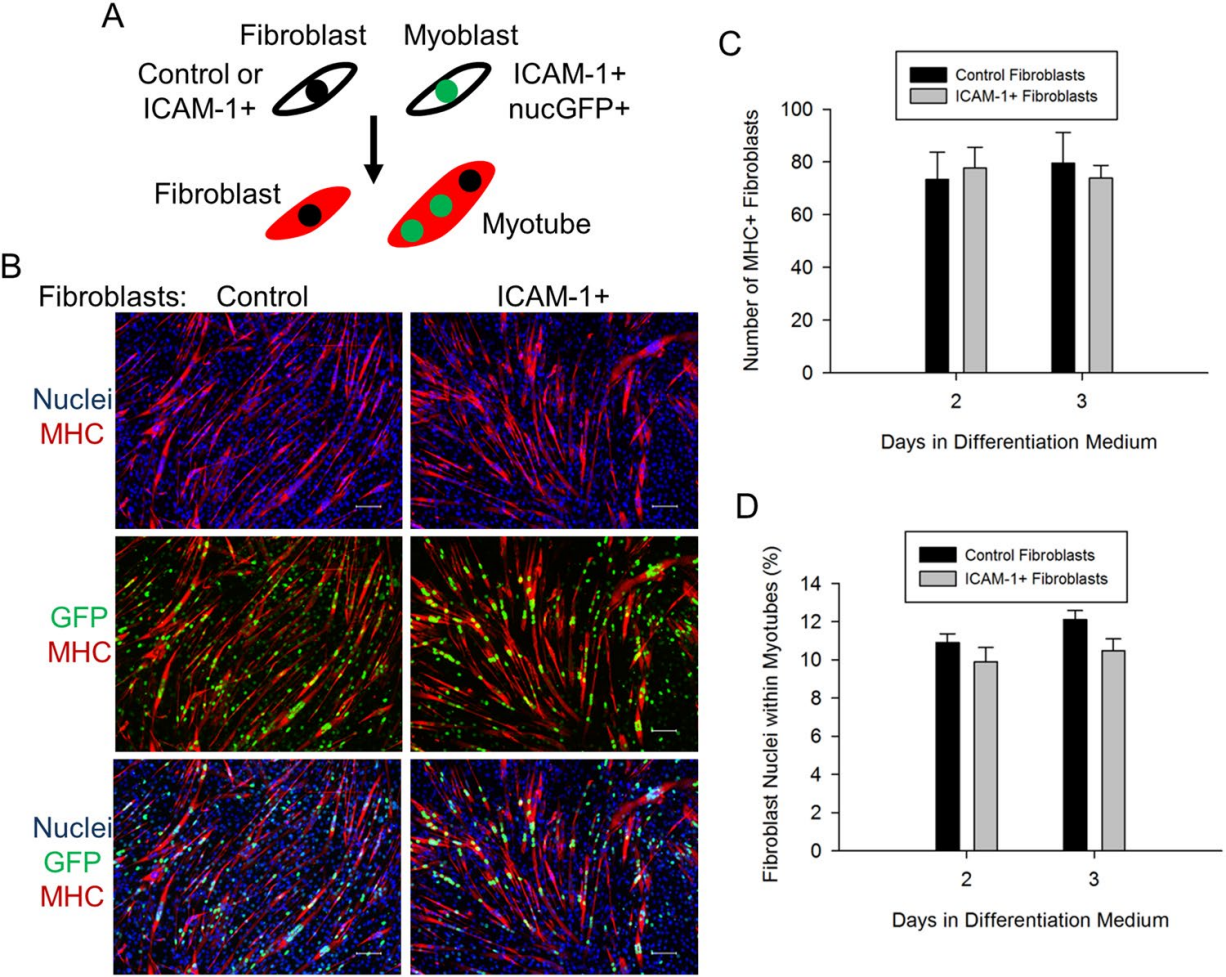
ICAM-1-ICAM-1 interactions in the myogenic conversion of fibroblasts. (**A**) Control or ICAM-1+ fibroblasts were mixed in equal number with ICAM-1+ nucGFP+ myoblasts and the number of fibroblasts expressing MHC (red) and the number of fibroblast nuclei within myotubes were quantified. (**B**) Images of DAPI+ nuclei of fibroblasts and myoblasts (blue), MHC (red), and nuclei of ICAM-1+ nucGFP+ myoblasts (green) at 3 d of differentiation (scale bar = 100 µm). (**C**,**D**) Average number of MHC+ fibroblasts (**C**) and the percentage of fibroblast nuclei within myotubes (**D**). n = 6 replicates per group.

Fibroblasts do not constitutively express ICAM-1 (Figure S2)^28^ and lack the ability to fuse together. However, culturing fibroblasts with myoblasts causes a small fraction of fibroblasts to express myogenic markers (e.g., MHC) and fuse with myoblasts/myotubes through a mechanism involving cell-to-cell contact^29, 30^. We used the co-culture model to determine if ICAM-1-ICAM-1 interactions could augment the myogenic conversion of fibroblasts (Fig. 5A). Importantly, 50.4% of the total nuclei counted (n = 341,444) in co-cultures were fibroblast nuclei and the standard deviation in the percentage of fibroblasts at 2 and 3 d of differentiation was 2.9%.

In co-cultures containing control fibroblasts, 1.0% of the total number of fibroblasts expressed MHC (Fig. 5B and C), and 12% of the fibroblast nuclei were found within myotubes at 3 d of differentiation (Fig. 5D). At 3 d of differentiation, 64% of the myotubes contained at least one fibroblast nuclei. Forced expression of ICAM-1 by fibroblasts did not influence the number of MHC+ fibroblasts or the percentage of fibroblast nuclei within myotubes. No MHC was detected in cultures containing only control or ICAM-1+ fibroblasts. These findings demonstrate that the expression of ICAM-1 by fibroblasts does not augment their myogenic conversion when cultured with ICAM-1+ myogenic cells.

### Cytoplasmic Domain of ICAM-1 Contributes to ICAM-1 Mediated Myoblast Fusion

**Figure 6:**
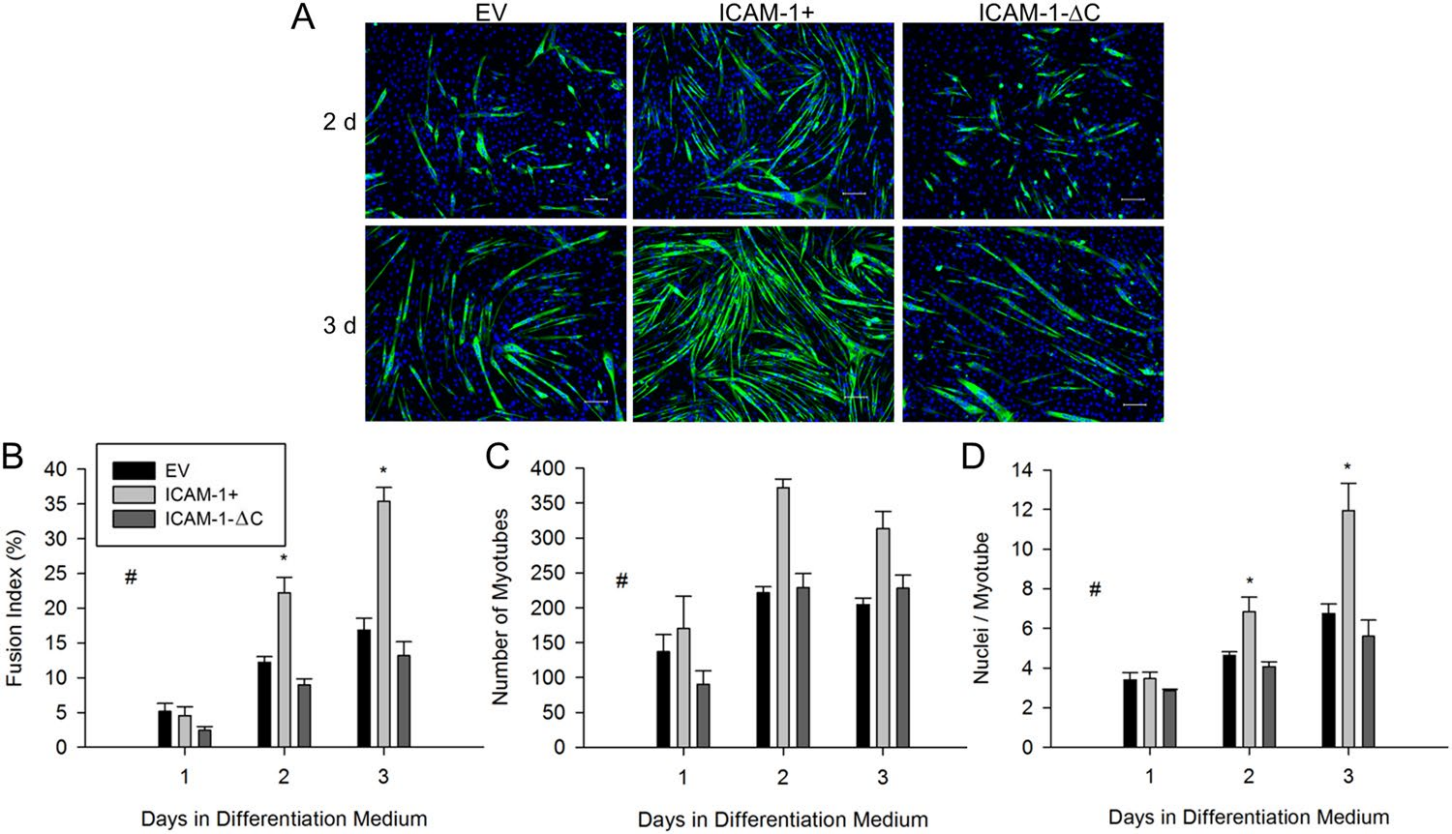
Cytoplasmic domain of ICAM-1 in myoblast fusion. (**A**) Images of MHC (green) and nuclei (blue) in EV, ICAM-1+, and ICAM-1-∆C cells at 2 and 3 d of differentiation (scale bar = 100 µm). (**B–D**) Fusion index (**B**), myotube number (**C**), and average number of nuclei within myotubes (**D**). ^#^Higher for ICAM-1+ compared to EV and ICAM-1-∆C cells throughout 3 d of differentiation (main effect for cell line; p < 0.001). *Higher for ICAM-1+ compared to EV and ICAM-1-∆C cells at indicated day of differentiation (interaction effect; p < 0.003). n = 4–6 replicates per group.

We previously demonstrated that peptide inhibition of the cytoplasmic domain of ICAM-1 reduced myotube formation and myonuclear accretion in ICAM-1+ myoblasts^4^. Consistent with these findings^4^, genetic deletion of the cytoplasmic domain of ICAM-1 returned indices of myoblast fusion to control levels (Fig. 6). Taken together, our findings demonstrate that ICAM-1 mediated adhesive interactions between opposing myoblasts augments their fusion through the signaling function of the cytoplasmic domain of ICAM-1.

### Homophilic Binding of ICAM-1 Promotes Lamellipodia and Spreading in Myoblasts

**Figure 7:**
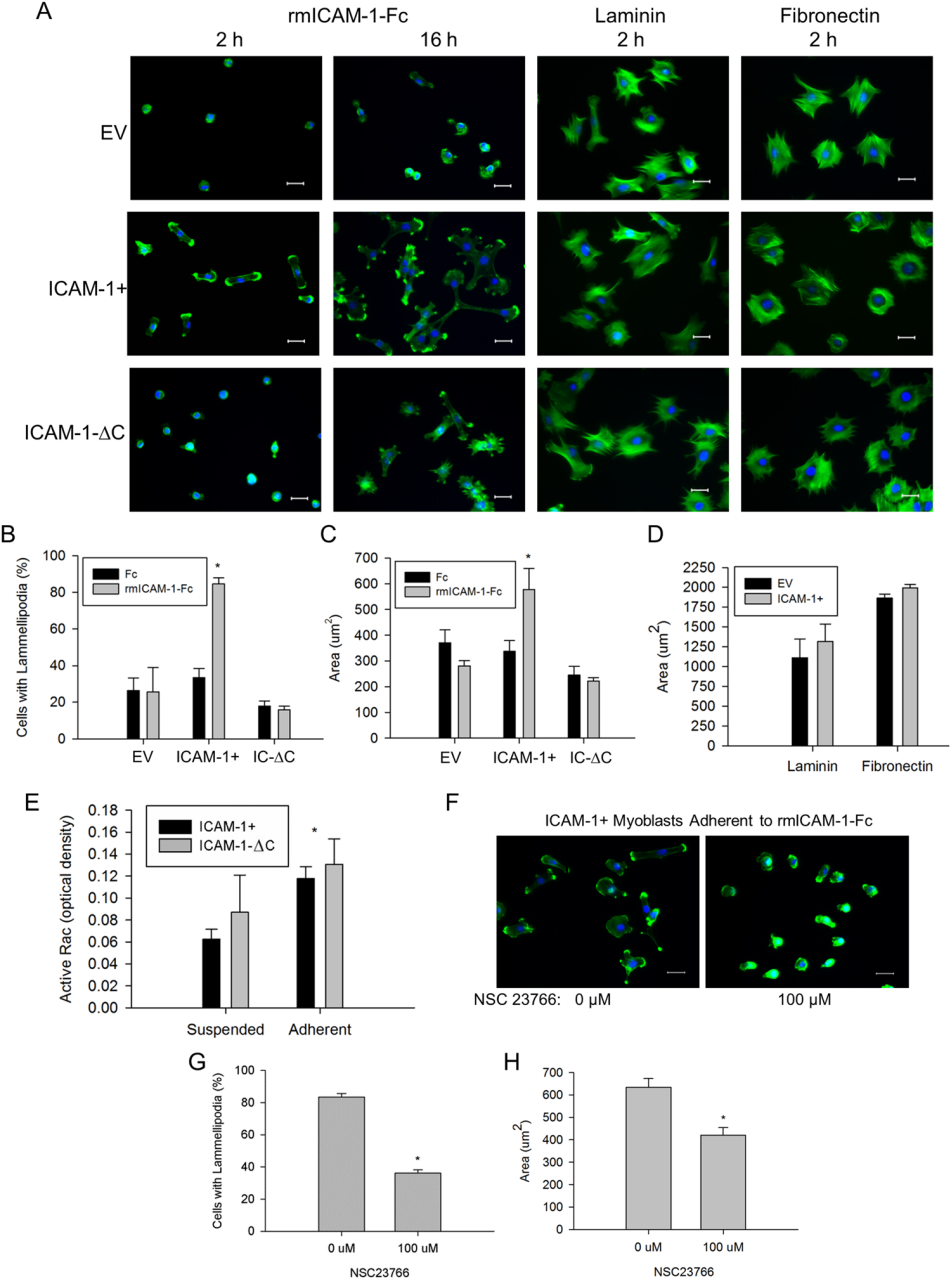
Adhesion-induced alterations in the actin cytoskeleton and Rac activity. (**A**) Images of F-actin (green) and nuclei (blue) in myoblasts adherent to rmICAM-1-Fc, laminin, or fibronectin (scale bar = 25 µm). (**B**) Percentage of cells with one of more prominent lamellipodium 2 h after myoblasts to wells coated with hIgG-Fc (Fc) and rmICAM-1-Fc. *Higher for ICAM-1+ compared to EV and ICAM-1-∆C myoblasts (interaction effect; p < 0.001). n = 4 replicates per group. A total of 149–406 myoblasts for each cell line were analyzed. (**C**) Cytoplasmic area 2 h after adding myoblasts to Fc and rmICAM-1-Fc coated wells. A total of 154–326 myoblasts for each cell line were analyzed. *Higher for ICAM-1+ compared to EV and ICAM-1-∆C myoblasts (interaction effect; p < 0.001). n = 4 replicates per group. (**D**) Cytoplasmic area 2 h after adding myoblasts to laminin and fibronectin coated wells. n = 4 replicates per group. A total of 261–291 myoblasts for each cell line were analyzed. (**E**) Active Rac in ICAM-1+ and ICAM-1-∆C myoblasts in suspension and adherent to rmICAM-1-Fc. *Higher for adherent compared to suspended myoblasts (main effect; p < 0.05). n = 4 replicates per group. (**F**) Images of F-actin (green) and nuclei (blue) of ICAM-1+ myoblasts adherent to rmICAM-1-Fc after a 2 treatment with NSC23766. (**G**) Percentage of cells with one of more prominent lamellipodium after a 2 treatment with NSC23766. A total of 583–604 ICAM-1+ myoblasts were analyzed. *Lower for 100 µM compared to 0 µM (p < 0.001). n = 6 replicates per group. (**H**) Cytoplasmic area for ICAM-1+ myoblasts adherent to rmICAM-1-Fc after a 2 h treatment with NSC23766. A total of 365–495 ICAM-1+ myoblasts were analyzed. *Lower for 100 µM compared to 0 µM (p < 0.003). n = 6 replicates per group.

As adhesion-induced remodeling of the actin cytoskeleton and myoblast fusion are dependent on polymerization of globular (G-) to filamentous (F-) actin^3, 8, 31^, we examined the localization of F-actin in fusion competent myoblasts 2 and 16 h after adding them to rmICAM-1-Fc coated wells. For ICAM-1+ myoblasts, F-actin was primarily localized to fan-like membrane protrusions called lamellipodia, which in many cases were apparent at both ends of elongated myoblasts (Fig. 7A). The percentage of cells that had one or more prominent lamellipodium 2 h after adding them to rmICAM-1-Fc coated wells was higher for ICAM-1+ compared to EV and ICAM-1-∆C myoblasts (Fig. 7B). For ICAM-1+ myoblast adherent to rmICAM-1-Fc coated wells, the number of lamellipodium per cell and the size of lamellipodia appeared to increase over time. Filopodia (finger-like membrane protrusions) were rarely observed in ICAM-1+ myoblasts and were prevalent in ICAM-1-∆C myoblasts at 16 h, even in the absence of discernible lamellipodia. These observations indicate that homophilic binding of ICAM-1 elicits lamellipodia enriched foci of F-actin in myoblasts through the cytoplasmic domain of ICAM-1.

Bundles of F-actin/stress fibers and cortical F-actin filaments were lacking in EV, ICAM-1+ and ICAM-1-∆C myoblasts adherent to rmICAM-1-Fc, and were prevalent 2 h after adding them to wells coated with laminin and fibronectin. Furthermore, lamellipodia were not prevalent in EV, ICAM-1+ and ICAM-1-∆C myoblasts adherent to laminin and fibronectin. These observations demonstrate that homophilic binding of ICAM-1 elicits actin-based membrane and cytoskeletal changes in ICAM-1+ myoblasts that are distinctly different from their adhesion to laminin and fibronectin^31, 32^.

Cytoplasmic area of myoblasts was quantified 2 h after adding them to coated wells and used as readout of actin-based cell spreading. Cytoplasmic area was ~2 fold higher for ICAM-1+ myoblasts adherent to rmICAM-1-Fc compared to Fc, and compared to ICAM-1-∆C and EV myoblasts adherent to rmICAM-1-Fc (Fig. 7C). This finding demonstrates that homophilic binding of ICAM-1 augments myoblast spreading through the cytoplasmic domain of ICAM-1.

As expected, the cytoplasmic area was similar for EV and ICAM-1+ myoblasts adherent to fibronectin and laminin (Fig. 7D). The cytoplasmic area was numerically higher for ICAM-1+ myoblast adherent to fibronectin and laminin compared to rmICAM-1-Fc, which further substantiates the specificity of ICAM-1-ICAM-1 interactions in eliciting changes in the actin cytoskeleton.

We interpret our findings that the cytoplasmic domain of ICAM-1 contributes to ICAM-1 mediated lamellipodia and spreading in myoblasts without influencing the adhesive function of the extracellular domain of ICAM-1 (see Fig. 1F and G) to indicate that ICAM-1 signaling resulting from homophilic binding of ICAM-1 elicits membrane and cytoskeletal changes necessary for myoblast fusion.

### Homophilic Binding of ICAM-1 Increases Rac Activity in Myoblasts

Cell adhesion initiates actin-based lamellipodia, filopodia, and/or stress fiber formation through activation of Rho GTPases Rac, cdc42, and RhoA, respectively^33^. As lamellipodia were the prevailing morphological feature of ICAM-1+ myoblasts adherent to rmICAM-1-Fc, we tested the hypothesis that ICAM-1-ICAM-1 adhesive interactions augment Rac activity in myoblasts through the cytoplasmic domain of ICAM-1. Active Rac (GTP bound to all 3 isoforms of Rac, not just Rac1) was quantified via G-LISA in fusion competent myoblasts that were collected at low confluence, and maintained in suspension or resuspended in differentiation medium and added to wells coated with rmICAM-1-Fc. We did not measure active Rac in EV myoblasts because of their poor adhesion to wells coated with rmICAM-1-Fc (see Fig. 1D and E). Active Rac was similar in cell suspensions of ICAM-1+ and ICAM-1-∆C myoblasts, and 47% higher for ICAM-1+ myoblasts adherent to rmICAM-1-Fc compared to suspended ICAM-1+ myoblasts (Fig. 7E). Unexpectedly, adhesion of ICAM-1-∆C myoblasts to rmICAM-1-Fc increased active Rac to levels that were similar to those observed for ICAM-1+ myoblasts adherent to rmICAM-1-Fc. These findings indicate that ICAM-1-ICAM-1 interactions augment Rac activity in myoblasts through a mechanism that is not dependent on the cytoplasmic domain of ICAM-1.

### Inhibition of Rac Reduces ICAM-1 Mediated Lamellipodia and Spreading in Myoblasts

The involvement of Rac in membrane and cytoskeletal changes resulting from ICAM-1-ICAM-1 interactions was tested by inhibiting Rac activity in ICAM-1+ myoblasts adherent to rmICAM-1-Fc using NSC23766 (100 µM) (Fig. 7F), which blocks the binding of guanine nucleotide exchange factors (GEFs) TrioN and Tiam1 with Rac^34^. Inhibition of Rac reduced lamellipodia formation (Fig. 7G) and cytoplasmic area (Fig. 7H) 2 h after adding ICAM-1+ myoblasts to rmICAM-1-Fc coated wells. Thus, Rac signaling appears to serve as a mechanism through which homophilic binding of ICAM-1 mediates lamellipodia and spreading in ICAM-1+ myoblasts.

### ICAM-1 Augments Rac Activity During Myoblast Fusion

**Figure 8:**
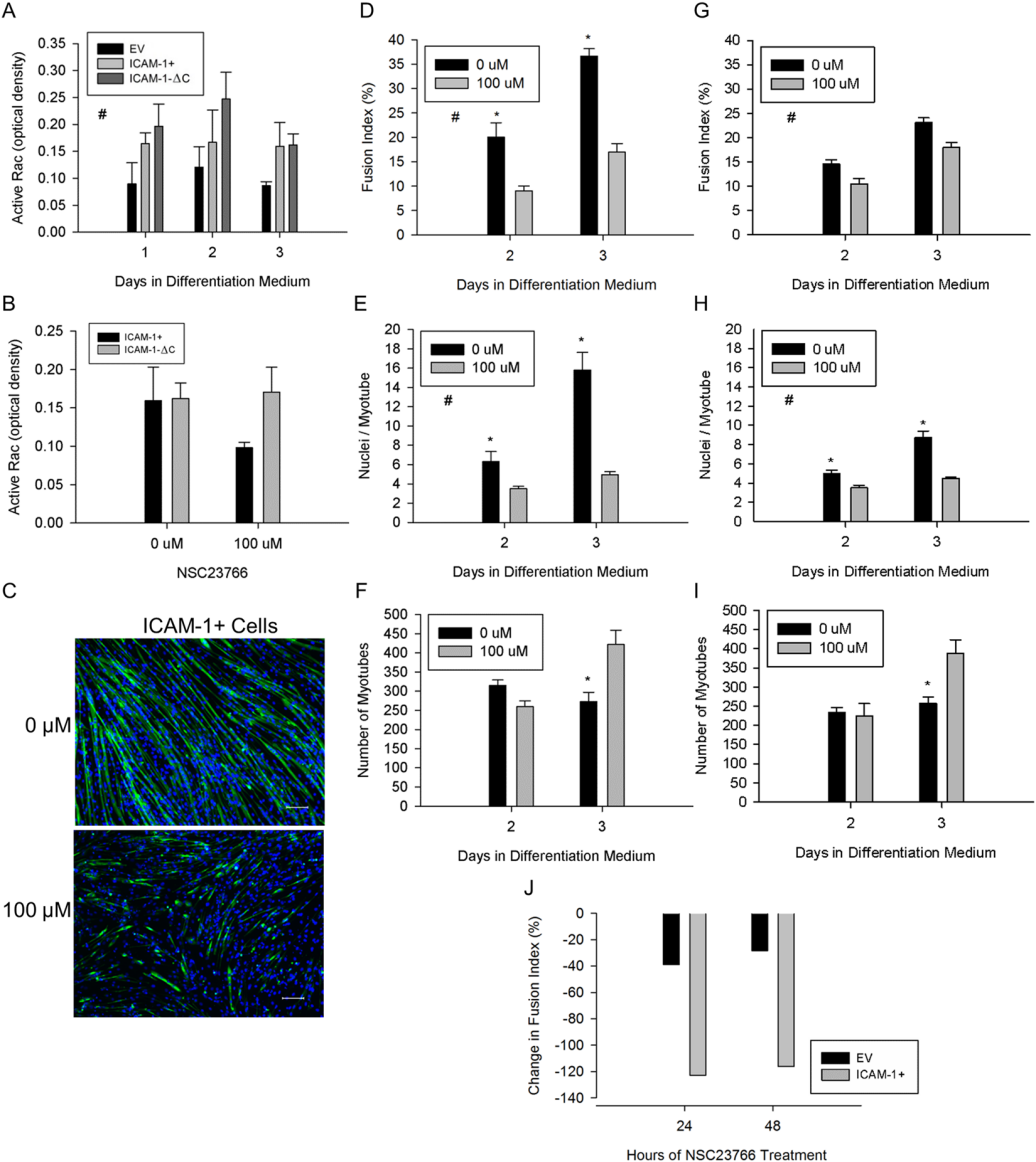
Rac activity in ICAM-1 mediated myoblast fusion. (**A**) Active Rac through 3 d of differentiation. ^#^Higher for ICAM-1+ and ICAM-1-∆C compared to EV cells throughout 3 d of differentiation (main effect for cell line; p = 0.004). n = 4 replicates per group. (**B**) Active Rac after treating ICAM-1+ and ICAM-1-∆C cells at 1 d of differentiation with NSC23766 (100 µM) for 48 h. n = 4 replicates per group. (**C**) Representative images of MHC (green) and nuclei (blue) in ICAM-1+ cells after treating them at 1 d of differentiation with NSC23766 (100 µM) for 48 h. (**D–F**) Fusion index (**D**), average number of nuclei within myotubes (**E**), and myotube number (**F**) in ICAM-1+ cells after a 24 or 48 h treatment with NSC23766 (100 µM). ^#^Significant (p < 0.001) main effect for concentration of NSC23766. *Significant (p < 0.05) interaction effect at specified day of differentiation. n = 4–6 replicates per group. (**G**–**I**) Fusion index (**G**), average number of nuclei within myotubes (**H**), and myotube number (**I**) in EV cells after a 24 or 48 h treatment with NSC23766 (100 µM). ^#^Significant (p < 0.001) main effect for concentration of NSC23766. *Significant (p < 0.05) interaction effect at specified day of differentiation. n = 4–6 replicates per group. (**J**) Percent change in the group mean for fusion index in EV and ICAM-1+ cells treated with NSC23766 (100 µM).

As prior studies have implicated adhesion-induced Rac signaling as a mechanism for myoblast fusion^9, 11, 35–37^, we quantified active Rac (i.e., GTP bound to Rac1, 2, and/or 3) in EV, ICAM-1+ and ICAM-1-∆C cells through 3 d of differentiation. Active Rac was 1.7 fold higher in ICAM-1+ compared to EV through 3 d of differentiation (Fig. 8A). Levels of active Rac were similar between ICAM-1+ and ICAM-1-∆C cells through 3 d of differentiation. These findings indicate that ICAM-1 augments Rac activity during myoblast fusion through a mechanism that is not dependent on the cytoplasmic domain of ICAM-1.

To further explore Rac activity during myoblast fusion, we quantified active Rac in ICAM-1+ and ICAM-1-∆C cells after treating them at 1 d of differentiation with NSC23766 (100 µM) for 48 h. NSC23766 reduced active Rac in ICAM-1+ cells by 62%; whereas, it increased active Rac by 5% in ICAM-1-∆C cells (Fig. 8B; p-value for interaction = 0.25). As NSC23766 blocks the binding of TrioN and Tiam1 with Rac^34^, TrioN and/or Tiam1 appear to contribute to the activation of Rac in ICAM-1+, but not ICAM-1-∆C cells during myoblast fusion.

### Inhibition of Rac Reduces ICAM-1 Mediated Myogenic Cell Fusion

We treated cultures with NSC23766 (100 µM) at 1 d of differentiation for 24 or 48 h to determine the extent to which activation of Rac contributes to ICAM-1-mediated myotube formation and myonuclear accretion. Rac inhibition in ICAM-1+ cells reduced fusion index and the average number of nuclei with myotubes by 2–2.5 fold (Fig. 8C–E). The number of myotubes at 2 d of differentiation was reduced by 25% and increased by 35% at 3 d of differentiation by Rac inhibition (Fig. 8F), suggesting that activation of Rac contributes to ICAM-1 mediated myoblast-myoblast and myotube-myotube fusion. Similar changes in myotube indices were observed in EV cells treated with NSC23766 (Fig. 8G–I). Importantly, the magnitude of change in the fusion index (Fig. 8J) and myonuclear number (data not reported) with Rac inhibition were 2–4 fold greater for ICAM-1+ compared to EV cells, which is consistent with the nearly 2 fold higher levels of active Rac in ICAM-1+ cells. Thus, ICAM-1 mediated myoblast and myotube fusion appears to be attributable to enhanced activation of Rac.

### Homophilic Binding of ICAM-1 Facilitates Myotube Hypertrophy

We previously demonstrated that ICAM-1 expression by myofibers in overloaded muscles^19^ and myotubes *in vitro* ^4^ promotes their hypertrophy. As myonuclear accretion serves as a mechanism for hypertrophy of myotubes^1^, we quantified myonuclear number and myotube size in mixed cultures containing EV and ICAM-1+ nucGFP+ cells. At 2–5 d of differentiation, myotube area was 2–4 fold higher for myotubes that contained nuclei primarily from ICAM-1+ nucGFP+ myoblasts (>50% GFP+), compared to myotubes that contained nuclei primarily from EV myoblasts (≤50% GFP+) (Figure S4). Furthermore, the number of nuclei from ICAM-1+ nucGFP+ myoblasts within a myotube was highly correlated to myotube area (Figure S4; r = 0.95, n = 5337 myotubes). The correlational coefficient for the number of GFP+ nuclei within a myotube was higher than that observed for nuclei from EV myoblasts (r = 0.79, n = 5337 myotubes). These findings demonstrate that myonuclear accretion resulting from ICAM-1-ICAM-1 interactions facilitate myotube hypertrophy.

## Discussion

Communication between myoblasts and neighboring cells (e.g., myogenic cells, macrophages, and fibroblasts) through cell-to-cell adhesion and paracrine signaling is critically important in regenerative myogenesis as it orchestrates cellular and molecular processes that restore structure and function to injured muscle^1, 2, 17, 18, 38^. The development of therapeutic approaches to enhance such communication in skeletal muscle is hampered by limited knowledge of membrane structures that mediate adhesive and fusogenic properties of myoblasts. The present study addresses this significant gap in knowledge by elucidating mechanisms through which myogenic cell expression of ICAM-1 augments cell-to-cell interactions of myogenesis. Evidence presented herein demonstrates that ICAM-1 enhanced myoblast adhesion to myoblasts and myotubes through homophilic *trans*-interactions. Such adhesive interactions triggered an increase in active Rac, actin-based lamellipodia and spreading in myoblasts, as well as myoblast fusion and myotube hypertrophy. Our novel findings provide mechanistic support for a paradigm in which induced expression of ICAM-1 by myogenic cells augments cell-to-cell interactions of regenerative myogenesis.

A major finding of the present study was that homophilic *trans*-interactions served as a mechanism through which ICAM-1 augmented myoblast adhesion to myoblasts and myotubes. Homophilic binding of the extracellular domain of ICAM-1 was demonstrated through the use of rmICAM-1-Fc, which was found to bind to ICAM-1 expressed by myoblasts, as well as serve as a substratum for their adhesion. Homophilic *trans*-interactions for ICAM-1 were also observed when ICAM-1+ myoblasts were mixed with myoblasts that do not express ICAM-1 (i.e., EV myoblasts). Importantly, myoblast adhesion resulting from ICAM-1-ICAM-1 interactions was not dependent on the signaling function of the cytoplasmic domain of ICAM-1. Our findings complement those of Barreiro *et al*.^39^, who reported ICAM-1-ICAM-1 interactions in cultured endothelial cells.

Prior studies have used cytoplasmic localization of fluorescent proteins (e.g., GFP) or chemicals (e.g., cell tracking dyes) in cell mixing experiments to determine the extent to which membrane proteins mediate myoblast fusion^6, 8^. In the present study, we used a cell mixing approach in which GFP was localized to the nucleus of ICAM-1+ myoblasts to quantify the extent to which homophilic *trans*-interactions for ICAM-1 influence myotube formation and myonuclear accretion. We report that myotube number was greater when ICAM-1+ myoblasts fused with each other, compared to the fusion of EV myoblasts with EV myoblasts. Furthermore, myonuclear number was higher for myotubes that contained nuclei from only ICAM-1+ myoblasts, compared to myotubes that contained nuclei from only EV myoblasts. Importantly, ICAM-1 mediated myoblast fusion was dependent on adhesion-induced ICAM-1 signaling, as antibody neutralization of the extracellular domain of ICAM-1^4^, as well as peptide^4^ and genetic (present study) inhibition of the cytoplasmic domain of ICAM-1, reduced indices of myoblast fusion to control levels. Collectively, our findings demonstrate that myoblast adhesion to myoblasts and myotubes through ICAM-1-ICAM-1 interactions augments myoblast fusion through the signaling function of the cytoplasmic domain of ICAM-1.

We explored the extent to which ICAM-1 expression by fibroblasts augments their fusion with myogenic cells for several reasons. One, fibroblasts fuse with myogenic cells *in vitro* through a mechanism involving cell-to-cell contact/adhesion^29, 30^. Two, culturing myoblasts with fibroblasts is an excellent model to study the fusogenic property of membrane proteins^8, 40^. Given these reasons, as well as our finding that homophilic *trans*-interactions for ICAM-1 augmented myogenesis (present study), we hypothesized that fibroblast adhesion to myoblasts and myotubes through ICAM-1-ICAM-1 interactions would augment their myogenic conversion. Contrary to our hypothesis, ICAM-1 expression by fibroblasts failed to augment their fusion with ICAM-1+ myogenic cells, which suggests that fusion resulting from ICAM-1-ICAM-1 interactions is restricted to cells of the myogenic lineage.

We have begun to identify mechanisms through which ICAM-1 signaling augments myogenesis. Our prior work demonstrated that ICAM-1 augmented myotube formation and myonuclear accretion through a mechanism that is independent of myoblast differentiation and p38α signaling^4^, and our current findings indicate that ICAM-1 augments the fusogenic property of myoblasts through adhesion-induced activation of Rac, and a subsequent increase in actin-based membrane and cytoskeletal dynamics. Specifically, ICAM-1-ICAM-1 interactions augmented levels of active Rac during myoblast adhesion and fusion, as well as triggered lamellipodia, spreading, and fusion of myoblasts. Our findings are consistent with a role of Rac in regulating lamellipodia dynamics in other cell types^33, 41, 42^, and studies that demonstrated that genetic and/or chemical inhibition of Rac impairs myoblast fusion^11, 43, 44^. Although Rac is involved in the regulation of membrane dynamics during cell migration^33^, ICAM-1 did not influence myoblast motility or their migration towards other myoblasts. We speculate that Rac-mediated lamellipodia dynamics resulting from ICAM-1-ICAM-1 interactions increased the area of adhesion between opposing myogenic cells, which in turn augmented membrane and cytoskeletal changes necessary for membrane union.

ICAM-1 is capable of activating intracellular signaling molecules and reorganizing the actin cytoskeleton upon ligation of the extracellular domain of ICAM-1^45, 46^. In endothelial cells, antibody binding to ICAM-1 activates small GTPases (RhoA, RhoG, Rac, and cdc42) and the formation of cup-like docking structures for leukocytes^47–51^. Such changes have been attributed to the ability of the cytoplasmic domain of ICAM-1 to bind GEFs (e.g., TrioN, Ect2, LARG, and SGEF) and actin-binding proteins (e.g., ezrin, α-actinin, filamins, cortactin, and F-actin)^46–48, 51, 52^. Antibody binding to ICAM-1 also causes ICAM-1 to associate with Src family of kinases within lipid rafts, which are capable of activating GEFs^46, 53, 54^. Our finding that NSC23766 reduced active Rac, lamellipodia, and spreading in ICAM-1+ myoblasts is consistent with the ability of the cytoplasmic domain of ICAM-1 to bind TrioN^47^ and regulators of the actin cytoskeleton^46^. However, genetic deletion of the cytoplasmic domain of ICAM-1, and treatment of ICAM-1-∆C cells with NSC23766 failed to reduce elevated levels of active Rac. As ICAM-1-∆C myoblasts cells showed impairments in lamellipodia, spreading, and fusion, it’s conceivable that the downstream effectors of Rac^55^ in ICAM-1-∆C myoblasts are different from those in ICAM-1+ myoblasts. Further study is needed to determine the mechanisms through which ICAM-1-ICAM-1 interactions regulate Rac activity in myogenic cells.

Based on our current and prior work^4, 19, 20^, we propose that the induced expression of ICAM-1 by myogenic cells augments regenerative and hypertrophic processes in skeletal muscle after increased use and/or injury. Evidence presented herein provides mechanistic support for such a paradigm by demonstrating that ICAM-1 augments the adhesive and fusogenic properties of myoblasts through homophilic *trans*-interactions and adhesion-induced, and Rac-mediated remodeling the actin cytoskeleton. The myonuclear accretion resulting from ICAM-1-ICAM-1 interactions also facilitated myotube hypertrophy, which occurs through a mechanism involving the signaling function of the cytoplasmic domain of ICAM-1, Akt/p70s6k signaling, and protein synthesis^4^. A defined role of ICAM-1 in regenerative myogenesis however, awaits future studies that reveal the specific contribution of myogenic cell expression of ICAM-1 to regenerating myofiber formation and hypertrophy after increased muscle use and/or injury. We speculate that myogenic cell expression of ICAM-1 would complement the actions of other membrane proteins that mediate regenerative and hypertrophic processes within skeletal muscle (e.g., myomaker)^8, 40, 56^. Results from the present and future studies will help define novel therapeutic therapies that restore and/or enhance the structure and function of skeletal muscle.

## Materials and Methods

### Transfections

Populations of C2C12 myoblasts (ATCC) and 10T1/2 fibroblasts (ATCC) were transfected with a plasmid containing murine ICAM-1 under transcriptional regulation of the human β-actin promoter (pHβAPr-1-ICAM-1)^57^ or with an empty vector (pHβAPr-1)^58^. Another population of myoblasts were co-transfected with the ICAM-1 plasmid and a plasmid containing histone2B linked to green fluorescent protein (H2B-GFP) (kindly provided by Richard Vallee at Columbia University). The sequence for the extracellular and transmembrane domains of murine ICAM-1 was inserted to the pHβAPr-1 vector and cloned to create a cell line of myoblasts lacking the cytoplasmic domain of ICAM-1. Transfections were performed using Lipofectamine^TM^ 2000 (Life Technologies)^4^. Transfection efficiency results are shown in Figure S3, as well as in our prior report^4^.

### Cell Cultures

Fusion competent myoblasts were generated by seeding myoblasts at high density (~20,000 cells/cm^2^) in Dulbecco’s modified eagle medium (DMEM; Thermo Scientific) containing 10% fetal bovine serum (Sigma-Aldrich; growth medium), allowing them to adhere to plates/dishes for 2 h, and then treating them with DMEM containing 2% horse serum (Sigma-Aldrich; differentiation medium) for 24 h^4, 6^. In cell mixing experiments, 2 cell lines (e.g., EV and ICAM-1+ nucGFP+ myoblasts) were mixed in equal number prior to seeding. Upon reaching 90% confluence, cells were treated with differentiation medium for up to 3 d.

### Coating of Cultureware

High binding 96 well plates (Corning, Catalog #3361) and 6 well non-treated plates (Thermo Scientific, Catalog #150239) were incubated overnight with 10 µg/ml of rmICAM-1-Fc (Biolegend) or 10 µg/ml of Fc (R&D Systems) in 0.1 M carbonate-bicarbonate buffer (pH 9.2). Wells were washed with phosphate buffered saline (PBS), blocked for 2 h in 0.1 M carbonate-bicarbonate buffer containing 1% BSA, and washed with PBS. Immunolabeling and western blotting for ICAM-1 confirmed the effectiveness of our coating protocol, as well as the dimeric state of rmICAM-1-Fc (Figure S5). In other experiments, 96 well tissue culture plates were incubated overnight with laminin (10 µg/ml; Sigma-Aldrich) or fibronectin (10 µg/ml; Sigma-Aldrich) in PBS, blocked for 2 h in 1% BSA, and washed with PBS.

### Myoblast Motility and Directed Migration

Fusion competent myoblasts at low confluence were analyzed for motility; whereas, their migration towards each other (directed migration) was determined using two chamber inserts (Ibidi). Cultures were placed in an incubation chamber (LiveCell™; Pathology Devices) and phase contrast images were captured every 5 min for 3 h (motility) or every 15 min for 20 h (directed migration).

Migratory paths of myoblasts that were visible throughout the time-lapse period were tracked using the manual track plug-in for Image J. The accumulated distance, velocity, displacement, and directionality of movement was determined using the chemotaxis and migration tool (Ibidi). Directed migration of myoblasts was also evaluated by the forward migratory index on the x-coordinate (FMI_x_), which reflects the efficiency of myoblast migration towards another population of myoblasts. A total of 80 fusion competent myoblasts for each cell line were analyzed for motility and directed migration in 3–4 independent experiments.

### Homophilic Binding of ICAM-1

Protein A coated magnetic beads (Invitrogen) were incubated for 1 h with a saturating amount of rmICAM-1-Fc (10 µg/50 µl of beads), washed with PBS containing 0.05% Tween-20 (PBS-T), and then cross-linked with 5 mM BS^3^ (Thermo Fisher Scientific). Beads were washed with 1 M glycine (pH 2.8) followed by PBS. Beads were then incubated overnight with rmICAM-1-Fc (10 µg), a non-chimeric form of rmICAM-1 (Stemcell Technologies; 10 µg)^59^, or cell lysates (300 µg of protein in RIPA buffer) and washed with PBS-T. Molecules bound to beads were eluted by suspending beads in 50 µl of Laemmli sample buffer containing TCEP (50 mM), and by heating (95 °C) samples for 5 min. Proteins within pulled-out fractions were separated using 10% SDS-PAGE gels (10 µl/gel) and ICAM-1 was detected via western blotting^4^.

### Myoblast Adhesion to Substratum

Fusion competent myoblasts were collected using StemPro® Accutase® (Invitrogen), and 100 µl of cells in differentiation medium were added to duplicate wells of 96 well plates (~9,000 cells/well). At 120 min of incubation (37 °C and 5% CO_2_), myoblasts were fixed using 70% methanol/30% acetone, and non-adherent cells were removed through PBS washes. Adherent myoblasts were permeabilized with 0.05% Triton X-100, and mounted in Fluoromount-G^TM^ containing the nuclear stain DAPI (SouthernBiotech).

Images of the entire well were captured using a 4X objective on an epifluorescence microscope (Olympus IX70) equipped with a CCD monochrome camera (RT KE SPOT^TM^; Diagnostic Instruments). Images were stitched together and the number of adherent cells/well was counted using image analysis software (Image Pro 7; Media Cybernetics). The number of adherent cells in 2 wells for each experimental condition was averaged. The mean number of myoblasts adherent to rhIgG1-Fc (N_Fc_), rmICAM-1-Fc (N_ICAM-1_), laminin, or fibronectin was expressed as a percentage of myoblasts that were added to each well (N_cells_). An adhesion index was calculated using the following equation: [(N_ICAM-1_ − N_Fc_)/N_cells_] × 100. Myoblast adhesion was quantified in 4 independent experiments for each substratum.

### Myoblast Aggregation

Fusion competent ICAM-1+ myoblasts were fluorescently labeled using CellTracker™ Green CMFDA (Life Technologies; 2.5 µM); whereas, fusion competent EV myoblasts were not fluorescently labeled. Cells were collected using StemPro® Accutase® (Invitrogen), suspended in differentiation medium (200 cells/µl), and ICAM-1+ and EV myoblasts were mixed in equal number in polypropylene tubes. Tubes were placed in a shaking water bath for 2 h and aliquots of cells were immobilized to slides using a cytospin centrifuge^4^. Cells were fixed in 4% formaldehyde, stained with WGA (Alexa Fluor® 350; Thermo Scientific), and mounted with Fluoromount-G^TM^ (SouthernBiotech). Images in six fields of view were captured using a 10X objective on an epifluorescence microscope.

The number of aggregates and the number of myoblasts within an aggregate were quantified using a macro function written for Image Pro 7 (Media Cybernetics). The number of ICAM-1+ cells within an aggregate was expressed as a percentage of the total number of WGA +cells within the aggregate. The number of EV cells was calculated by subtracting the total number of WGA+ cells from the number of ICAM-1+ cells within an aggregate. On average, ~2,000 myoblasts per slide were counted in 6 independent experiments.

### Myoblast Fusion

Cells were prepared for labeling^4^ and incubated overnight with one or more of the following antibodies: anti-sarcomeric myosin heavy chain (MHC; 1:20; clone MF20; Developmental Studies Hybridoma Bank) and anti-GFP AlexaFluor®488 (1:500; Thermo Fisher Scientific). Detection of bound MHC antibody was achieved through the use of an AlexaFluor®594 secondary antibody; whereas DAPI was used to detect nuclei. Images were captured in 9 standardized fields of view per well, with each field of view being separated by 200 µm.

Myotube indices in cultures containing only one cell line were quantified as previously described with minor modifications^4^. In cell mixing experiments, merged images of MHC, DAPI+ nuclei, and GFP+ nuclei were analyzed using a macro function written for Image Pro 7. Briefly, red (MHC), blue (nuclei), and green (nuclei of ICAM-1+ nucGFP+ cells) were extracted from a merged image to create separate luminance images. An outline of MHC+ cells was merged with images of GFP+ and DAPI+ nuclei and the number of nuclei within and outside the outline was counted.

A myotube was operationally defined as a MHC+ cell with 2 or more nuclei. The number of GFP+ nuclei within individual myotubes was expressed as a percentage of the total number of nuclei within the myotube. The number of nuclei from EV myoblasts or fibroblasts within individual myotubes was calculated by subtracting the total number of nuclei from the number of GFP+ nuclei within the myotube. This number was then expressed as a percentage of the total number of nuclei within the myotube. A fusion index was calculated for each cell type by expressing the number of nuclei within myotubes as a percentage of the total number EV, ICAM-1+ nucGFP+, or fibroblast nuclei. Indices of myotube size (e.g., area and maximum width) were quantified and used as measures of hypertrophy. Typically, 9–10,000 nuclei per well were analyzed. A total of 4–6 wells per experimental condition were analyzed in 3 or more independent experiments.

### Actin Cytoskeleton of Adherent Myoblasts

F-actin in fusion competent myoblasts was delineated using Alexa Fluor®488 conjugated phalloidin (Invitrogen) and nuclei were stained with DAPI. Ten images per well were captured using a 40X objective on an epifluorescence microscope. The area, length, and width of phalloidin stained myoblasts was quantified using Image Pro 7. Myoblasts that were in contact with each other or that had fused were excluded from our analysis. A total of 221–340 myoblasts for each experimental condition were analyzed in 4 independent experiments.

### Rac GTPase Assay

Active Rac in cell lysates was determined using the Rac G-LISA assay according to the manufacturer’s instructions (Cytoskeleton, Inc. Cat# BK125). In experiments examining adhesion-induced activation of Rac, fusion competent myoblasts at ~40% confluence were collected using StemPro® Accutase®. Cells (0.6 × 10^6^) were resuspended in Hanks balanced salt solution without calcium or magnesium (~50 cells/µl) and placed on ice for 30 min or resuspended in differentiation medium (300 cells/µl) and allowed to adhere to rmICAM-1-Fc coated wells for 2 h. Lysates of suspended and adherent cells, as well as cells treated with differentiation medium for up to 3 d were collected according to the manufacturer’s instructions. All lysates were normalized to a protein concentration of 0.5 µg/µl and analyzed in duplicate. The mean percent coefficient of variance for positive controls and cell lysates was 3.9% and 7.1%, respectively.

### Statistical Analyses

Data sets were analyzed using one or two way analysis of variance (ANOVA) using Sigma Stat statistical software (Systat). The Newman-Keuls *post*-*hoc* test was then used to locate differences between groups when the observed F ratio was statistically significant (p < 0.05). Data are reported as mean and standard error. The reported sample size for each dependent measure represents the number of replicates per group in 3 or more independent experiments. The Pearson product-moment correlation coefficient was calculated in correlational analyses.

## Electronic supplementary material

Supplemental Data
