# Supplementary material for: Intercellular adhesion molecule-1 augments myoblast adhesion and fusion through homophilic trans-interactions
Source: Sci Rep. 2017 Jul 11;7:5094. doi: 10.1038/s41598-017-05283-3 (PMC5506053; doi:10.1038/s41598-017-05283-3)
Supplement: Supplementary file 1 — Supplemental Data [file 41598_2017_5283_MOESM1_ESM.pdf]

Title: Intercellular adhesion molecule-1 augments myoblast adhesion and fusion through homophilic *trans*-interactions

Authors: Francis X. Pizza, Ryan A. Martin, Evan M. Springer, Maxwell S. Leffler, Bryce R. Woelmer, Isaac J. Recker, Douglas W. Leaman

Supplemental Figures

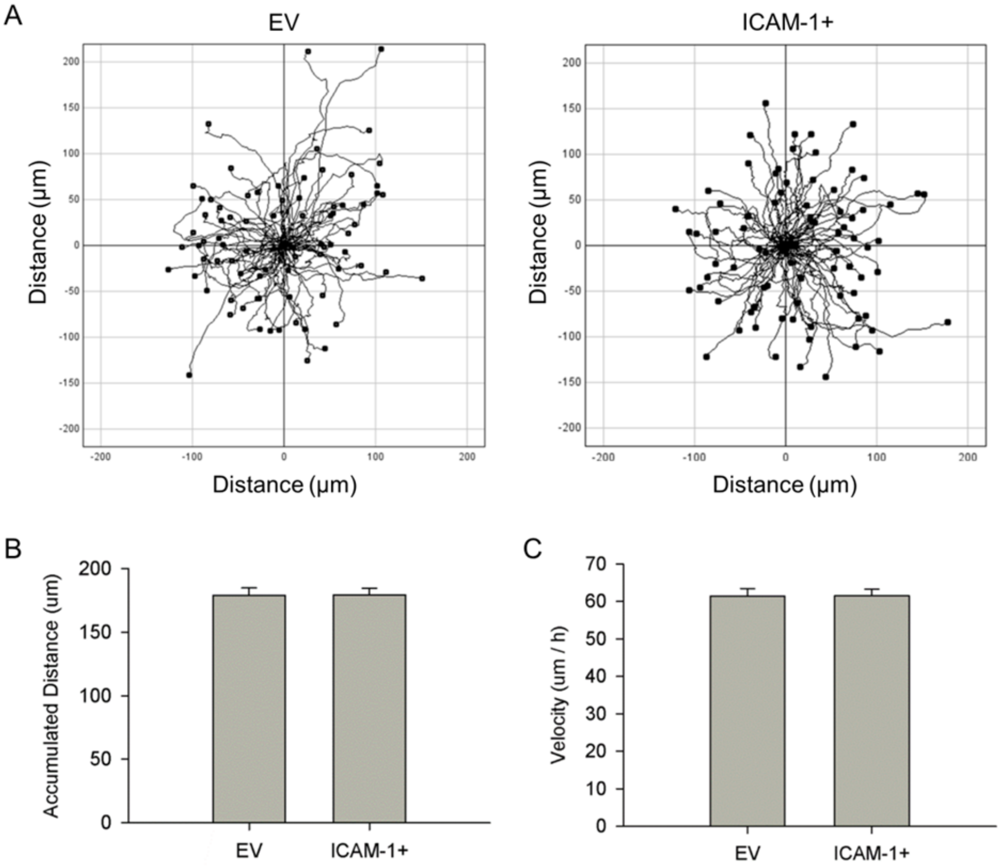

Figure S1. Myoblast motility. A) Migratory paths of EV and ICAM-1+ myoblasts for 3 h at 1 d of differentiation. B and C) Accumulated distance (B) traveled and velocity of movement (C) for EV and ICAM-1+ myoblasts. n=80 myoblasts per cell line in 4 independent experiments.

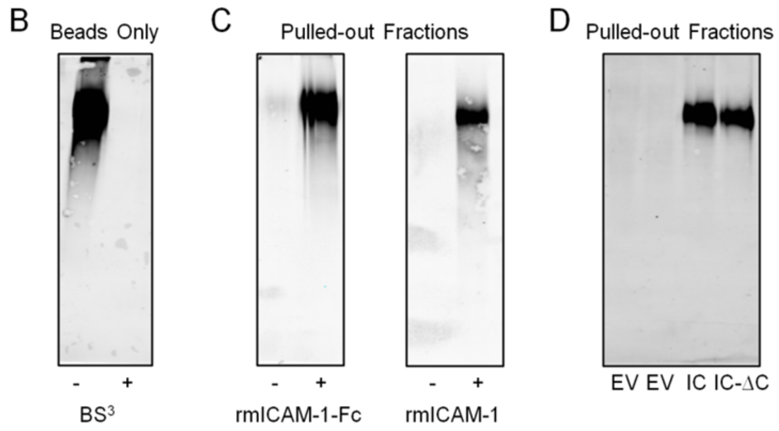

Figure S2. Western blot for ICAM-1 in beads coated with rmICAM-1-Fc. Images depict the entire lane (~200-25 kDa) for samples presented in Figure 1B, C and D. The ICAM-1 band appeared at ~110 kDa. B) BS^3 was used to covalently link rmICAM-1-Fc to beads. C) Beads crosslinked with ICAM-1 were incubated with PBS-T, rmICAM-1-Fc or rmICAM-1. ICAM-1 was detected in pulled-out fractions via western blotting. D) Western blots for ICAM-1 in pulled-out fractions of EV, ICAM-1+ (IC), and ICAM-1-ΔC (IC-ΔC) myoblasts.

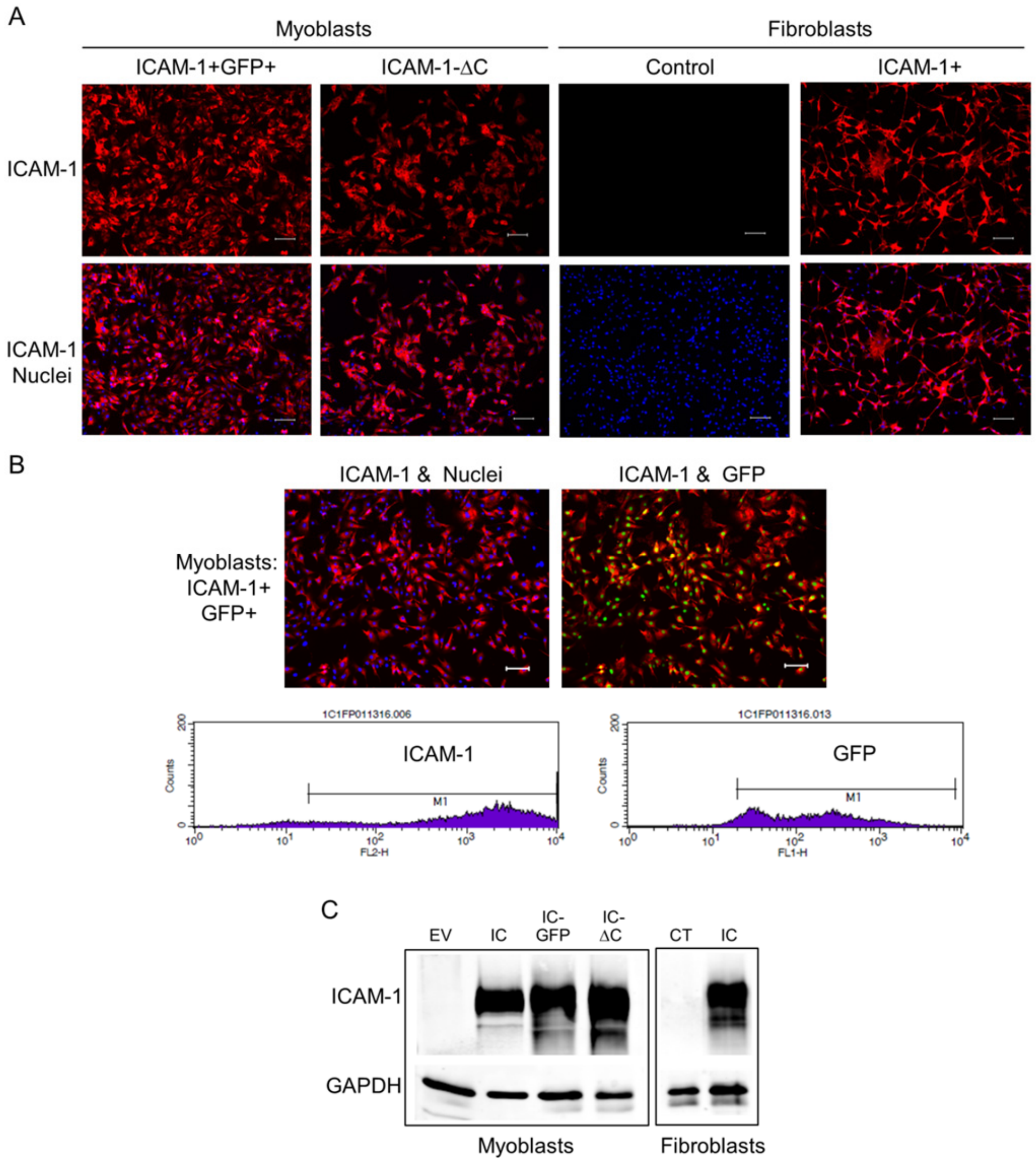

Figure S3. Transfection efficiency. A) Representative images of ICAM-1 (red) and nuclei (blue) in ICAM-1+GFP+ and ICAM-1-ΔC myoblasts, as well as control fibroblasts and ICAM-1+ fibroblasts (Scale bar = 100 µm). B) Representative images of GFP (green) and nuclei (blue) in ICAM-1+GFP+ myoblasts. Quantitative analysis revealed that 90-95% of ICAM-1+GFP+ myoblasts had nuclear localization of GFP (Scale bar = 100 µm). C) Representative western blot for ICAM-1 and GAPDH (loading control) in cell lines of myoblasts and fibroblasts. EV = empty vector, IC = ICAM-1+, IC-GFP = ICAM-1+nucGFP+, IC-ΔC = ICAM-1-ΔC, and CT = control fibroblasts.

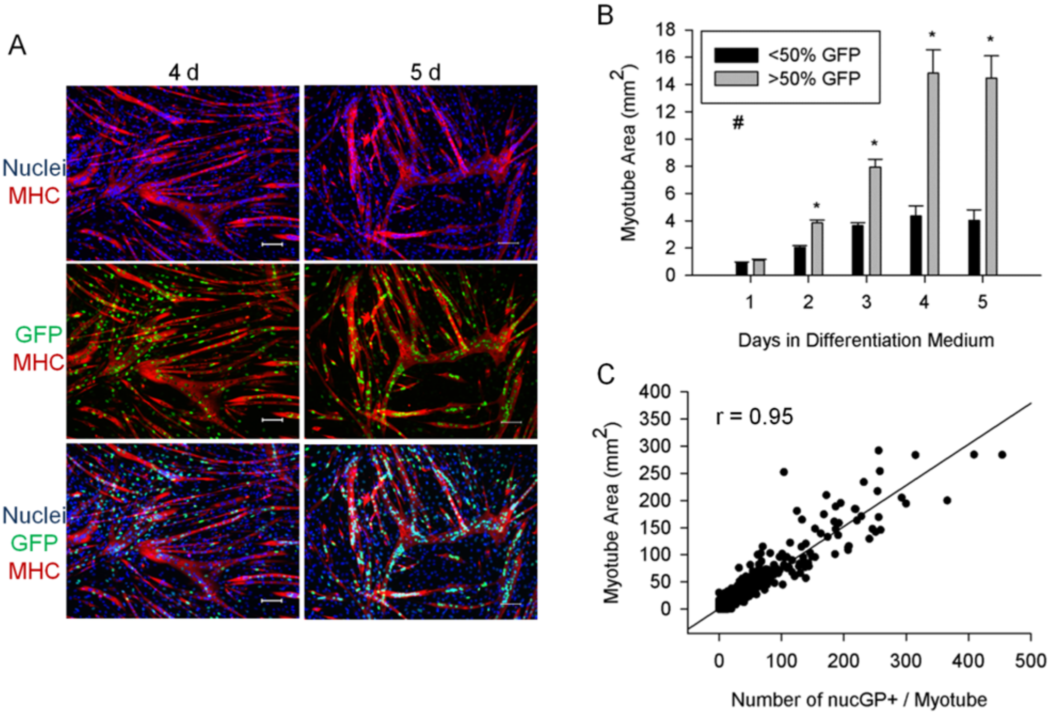

Figure S4. ICAM-1-ICAM-1 interactions in myotube hypertrophy. EV and ICAM-1+nucGFP+ myoblasts were mixed in equal number, and myotube area was quantified through 5 d of differentiation. A) Images of DAPI+ nuclei (blue) of EV and ICAM-1+nucGFP+ myoblasts, MHC (red), and nuclei of ICAM-1+nucGFP+ myoblasts (green) at 4 and 5 d of differentiation (scale bar = 100 μm). B) Quantitative analysis of myotube area for myotubes that contained nuclei primarily from ICAM-1+nucGFP+ (>50% GFP+; n=2827) or EV myoblasts (≤50% GFP+; n= 2944). # = higher for >50% GFP+ compared to ≤50% GFP+ throughout 5 d of differentiation (main effect for cell line; $p < 0.001$). * = higher for >50% GFP+ compared to ≤50% GFP+ at indicated day of differentiation (interaction effect; $p < 0.001$). n =4-6 replicates at each day of differentiation. C) Scatter plot of the number of number of GFP+ nuclei within a hybrid myotube at 2-5 d of differentiation vs. myotube area (n=5337 myotubes). A high Pearson-product moment correlation was observed (r=0.95; p<0.001).

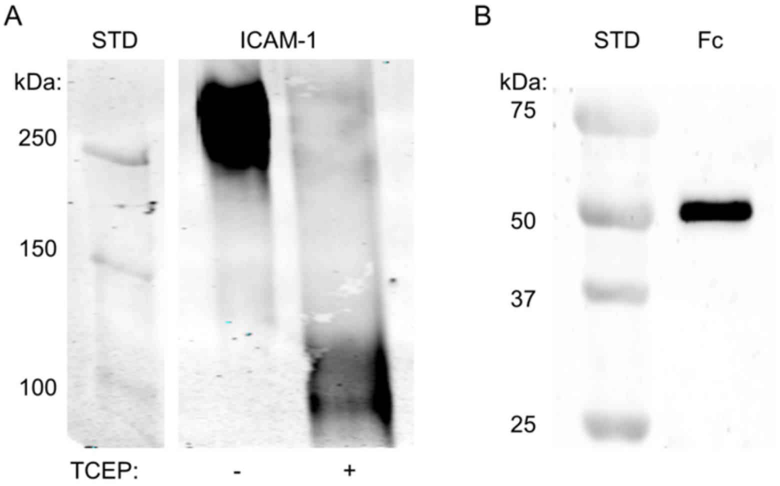

Figure S5. Wells coated with rmICAM-1-Fc and rhIgG1-Fc. A) Representative western blot for ICAM-1 in wells coated with rmICAM-1-Fc. rmICAM-1-Fc bound to wells was collected in non-reducing or reducing sample buffer, with TCEP serving as the reducing agent. Reducing conditions resulted in a large band shift in ICAM-1, demonstrating the dimeric state of rmICAM-1-Fc. B) Representative western blot for Fc in wells coated rhIgG1-Fc. Wells coated with rhIgG1-Fc were collected in non-reducing sample buffer.
